# 6-Chloro-3-[5-(4-fluoro­phen­yl)-1-phenyl-4,5-dihydro-1*H*-pyrazol-3-yl]-2-methyl-4-phenyl­quinoline

**DOI:** 10.1107/S1600536810000218

**Published:** 2010-01-09

**Authors:** Wan-Sin Loh, Hoong-Kun Fun, S. Sarveswari, V. Vijayakumar, B. Palakshi Reddy

**Affiliations:** a X-ray Crystallography Unit, School of Physics, Universiti Sains Malaysia, 11800 USM, Penang, Malaysia; b Organic Chemistry Division, School of Advanced Sciences, VIT University, Vellore 632 014, India

## Abstract

In the title compound, C_31_H_23_ClFN_3_, the pyrazole ring forms dihedral angles of 72.75 (7), 18.08 (9) and 86.26 (9)° with the quinoline ring system, the phenyl ring and the fluoro­phenyl ring, respectively. In the crystal, inter­molecular C—H⋯N hydrogen bonds link the mol­ecules into chains propagating along the *c* axis. The crystal structure is further stabilized by C—H⋯π inter­actions.

## Related literature

For a related structure and background to quinolines and pyrazolines, see: Loh *et al.* (2009 ▶). For the stability of the temperature controller used for the data collection, see: Cosier & Glazer (1986 ▶).
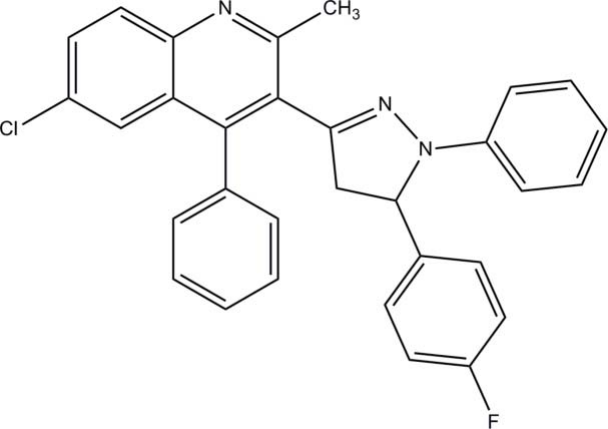


## Experimental

### 

#### Crystal data

C_31_H_23_ClFN_3_
*M* *_r_* = 491.97
Monoclinic, 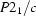

*a* = 9.4303 (2) Å
*b* = 28.2155 (6) Å
*c* = 9.6028 (2) Å
β = 106.636 (1)°
*V* = 2448.17 (9) Å^3^
*Z* = 4
Mo *K*α radiation
μ = 0.19 mm^−1^
*T* = 100 K
0.49 × 0.23 × 0.15 mm

#### Data collection

Bruker SMART APEXII CCD diffractometer
Absorption correction: multi-scan (*SADABS*; Bruker, 2009 ▶) *T*_min_ = 0.914, *T*_max_ = 0.973
38888 measured reflections
8947 independent reflections
6981 reflections with *I* > 2σ(*I*)
*R*_int_ = 0.031

#### Refinement

*R*[*F* ^2^ > 2σ(*F* ^2^)] = 0.067
*wR*(*F* ^2^) = 0.171
*S* = 1.08
8947 reflections
326 parameters
H-atom parameters constrained
Δρ_max_ = 0.78 e Å^−3^
Δρ_min_ = −0.25 e Å^−3^

### 

Data collection: *APEX2* (Bruker, 2009 ▶); cell refinement: *SAINT* (Bruker, 2009 ▶); data reduction: *SAINT*; program(s) used to solve structure: *SHELXTL* (Sheldrick, 2008 ▶); program(s) used to refine structure: *SHELXTL*; molecular graphics: *SHELXTL*; software used to prepare material for publication: *SHELXTL* and *PLATON* (Spek, 2009 ▶).

## Supplementary Material

Crystal structure: contains datablocks global, I. DOI: 10.1107/S1600536810000218/hb5303sup1.cif

Structure factors: contains datablocks I. DOI: 10.1107/S1600536810000218/hb5303Isup2.hkl

Additional supplementary materials:  crystallographic information; 3D view; checkCIF report

## Figures and Tables

**Table 1:** Hydrogen-bond geometry (Å, °) *Cg*1 and *Cg*2 are the centroids of the N1/C1/C2/C7–C9 and C10–C15 rings, respectively.

*D*—H⋯*A*	*D*—H	H⋯*A*	*D*⋯*A*	*D*—H⋯*A*
C15—H15*A*⋯N1^i^	0.93	2.57	3.493 (2)	173
C17—H17*A*⋯*Cg*1	0.97	2.86	3.6307 (19)	137
C31—H31*B*⋯*Cg*2^ii^	0.96	2.86	3.584 (2)	133

Symmetry codes: (i) 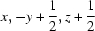; (ii) 
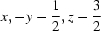
.

**Fig. 1.**
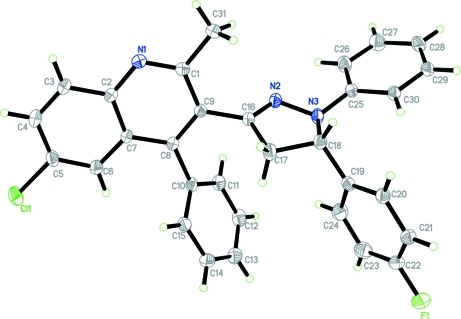
The molecular structure of (I), showing 50% probability displacement ellipsoids.

**Fig. 2.**
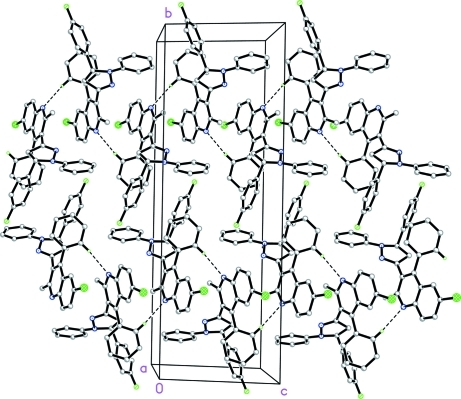
The crystal packing of (I), showing extended one-dimensional chains along the c axis. H atoms not involved in the intermolecular interactions (dashed lines) have been omitted for clarity.

## supplementary crystallographic information

### Comment

As part of our onging studies of substituted pyrazoline derivatives (Loh *et al.*,  2009), we now report the synthesis and structure of the title compound, (I).

The pyrazole ring (C16–C18/N2/N3) in (I) forms dihedral angles of 72.75 (7), 18.08 (9) and 86.26 (9) ° with the quinoline ring system (C1–C9/N1), phenyl (C25–C30) and fluorophenyl (C19–C24) rings, respectively. The quinoline ring system is approximately planar with a maximum deviation of 0.025 (2) Å at atom C9. Bond lengths and angles observed are comparable to a related structure (Loh *et al.*, 2009).

In the crystal packing, intermolecular C15—H15A···N1 hydrogen bonds link the molecules into extended one-dimensional chains along *c* axis. The crystal structure is further stabilized by C—H···π interactions.

### Experimental

A mixture of 1-(6-chloro-2-methyl-4-phenylquinolin-3-yl)-3-(4-flourophenyl) prop-2-en-1-one (0.001 *M*) and phenyl hydrazine in (0.007 *M*) in distilled methanol was refluxed for about 8 h. The resulting mixture was concentrated to remove methanol then poured on to ice and neutralized with diluted HCl. The resultant solid was filtered, dried and purified by column chromatography using 1:1 mixture of chloroform and petroleum ether. The compound was recrystallized from methanol to yield yellow blocks of (I). *M. p.*: 433–435 K, yield: 60%.

### Refinement

All hydrogen atoms were positioned geometrically [C–H = 0.93–0.98 Å] and were refined using a riding model, with *U*_iso_(H) = 1.2 or 1.5 *U*_eq_(C). A rotating group model was applied to the methyl group.

### Crystal data

**Table:**

C_31_H_23_ClFN_3_	*F*(000) = 1024
*M**_r_* = 491.97	*D*_x_ = 1.335 Mg m^−^^3^
Monoclinic, *P*2_1_/*c*	Mo *K*α radiation, λ = 0.71073 Å
Hall symbol: -P 2ybc	Cell parameters from 9957 reflections
*a* = 9.4303 (2) Å	θ = 2.3–31.9°
*b* = 28.2155 (6) Å	µ = 0.19 mm^−^^1^
*c* = 9.6028 (2) Å	*T* = 100 K
β = 106.636 (1)°	Block, yellow
*V* = 2448.17 (9) Å^3^	0.49 × 0.23 × 0.15 mm
*Z* = 4	

### Data collection

**Table:**

Bruker SMART APEXII CCD diffractometer	8947 independent reflections
Radiation source: fine-focus sealed tube	6981 reflections with *I* > 2σ(*I*)
graphite	*R*_int_ = 0.031
φ and ω scans	θ_max_ = 32.7°, θ_min_ = 2.3°
Absorption correction: multi-scan (*SADABS*; Bruker, 2009)	*h* = −14→14
*T*_min_ = 0.914, *T*_max_ = 0.973	*k* = −42→33
38888 measured reflections	*l* = −14→12

### Refinement

**Table:**

Refinement on *F*^2^	Primary atom site location: structure-invariant direct methods
Least-squares matrix: full	Secondary atom site location: difference Fourier map
*R*[*F*^2^ > 2σ(*F*^2^)] = 0.067	Hydrogen site location: inferred from neighbouring sites
*wR*(*F*^2^) = 0.171	H-atom parameters constrained
*S* = 1.08	*w* = 1/[σ^2^(*F*_o_^2^) + (0.0749*P*)^2^ + 1.6204*P*] where *P* = (*F*_o_^2^ + 2*F*_c_^2^)/3
8947 reflections	(Δ/σ)_max_ < 0.001
326 parameters	Δρ_max_ = 0.78 e Å^−^^3^
0 restraints	Δρ_min_ = −0.25 e Å^−^^3^

### Special details

**Table:**

Experimental. The crystal was placed in the cold stream of an Oxford Cyrosystems Cobra open-flow nitrogen cryostat (Cosier & Glazer, 1986) operating at 100.0 (1) K.
Geometry. All e.s.d.'s (except the e.s.d. in the dihedral angle between two l.s. planes) are estimated using the full covariance matrix. The cell e.s.d.'s are taken into account individually in the estimation of e.s.d.'s in distances, angles and torsion angles; correlations between e.s.d.'s in cell parameters are only used when they are defined by crystal symmetry. An approximate (isotropic) treatment of cell e.s.d.'s is used for estimating e.s.d.'s involving l.s. planes.
Refinement. Refinement of *F*^2^ against ALL reflections. The weighted *R*-factor *wR* and goodness of fit *S* are based on *F*^2^, conventional *R*-factors *R* are based on *F*, with *F* set to zero for negative *F*^2^. The threshold expression of *F*^2^ > σ(*F*^2^) is used only for calculating *R*-factors(gt) *etc*. and is not relevant to the choice of reflections for refinement. *R*-factors based on *F*^2^ are statistically about twice as large as those based on *F*, and *R*- factors based on ALL data will be even larger.

### Fractional atomic coordinates and isotropic or equivalent isotropic displacement parameters (Å^2^)

**Table:**

	*x*	*y*	*z*	*U*_iso_*/*U*_eq_	
Cl1	−0.17020 (5)	0.245611 (18)	0.84092 (5)	0.03203 (12)	
F1	0.83364 (15)	−0.06367 (4)	0.85393 (14)	0.0396 (3)	
N1	0.30003 (16)	0.28484 (5)	0.56387 (15)	0.0211 (3)	
N2	0.48193 (15)	0.15134 (5)	0.42869 (15)	0.0202 (3)	
N3	0.59506 (15)	0.11718 (5)	0.45432 (14)	0.0206 (3)	
C1	0.38175 (18)	0.25044 (6)	0.53450 (17)	0.0197 (3)	
C2	0.19192 (18)	0.27355 (5)	0.62820 (18)	0.0198 (3)	
C3	0.1078 (2)	0.31116 (6)	0.6618 (2)	0.0263 (3)	
H3A	0.1269	0.3422	0.6396	0.032*	
C4	−0.0011 (2)	0.30245 (6)	0.7265 (2)	0.0278 (4)	
H4A	−0.0551	0.3273	0.7494	0.033*	
C5	−0.03054 (19)	0.25528 (6)	0.75826 (19)	0.0235 (3)	
C6	0.04711 (18)	0.21773 (6)	0.72696 (18)	0.0211 (3)	
H6A	0.0248	0.1870	0.7484	0.025*	
C7	0.16176 (16)	0.22620 (5)	0.66153 (16)	0.0174 (3)	
C8	0.25181 (16)	0.18942 (5)	0.62996 (16)	0.0170 (3)	
C9	0.36189 (17)	0.20185 (5)	0.56766 (16)	0.0173 (3)	
C10	0.23459 (16)	0.13863 (5)	0.66548 (17)	0.0175 (3)	
C11	0.20683 (18)	0.10451 (6)	0.55481 (18)	0.0218 (3)	
H11A	0.1874	0.1139	0.4584	0.026*	
C12	0.20828 (19)	0.05650 (6)	0.5891 (2)	0.0263 (3)	
H12A	0.1870	0.0340	0.5154	0.032*	
C13	0.2413 (2)	0.04223 (6)	0.7329 (2)	0.0274 (4)	
H13A	0.2470	0.0101	0.7558	0.033*	
C14	0.26595 (19)	0.07580 (6)	0.8428 (2)	0.0244 (3)	
H14A	0.2863	0.0661	0.9391	0.029*	
C15	0.26037 (17)	0.12397 (6)	0.80953 (18)	0.0201 (3)	
H15A	0.2738	0.1464	0.8833	0.024*	
C16	0.47176 (17)	0.16606 (5)	0.55166 (17)	0.0179 (3)	
C17	0.58110 (18)	0.14337 (6)	0.68073 (17)	0.0230 (3)	
H17A	0.5326	0.1215	0.7301	0.028*	
H17B	0.6342	0.1670	0.7494	0.028*	
C18	0.68460 (17)	0.11731 (6)	0.60858 (17)	0.0193 (3)	
H18A	0.7747	0.1360	0.6195	0.023*	
C19	0.72498 (17)	0.06821 (6)	0.67002 (17)	0.0194 (3)	
C20	0.86967 (18)	0.05790 (6)	0.74894 (17)	0.0206 (3)	
H20A	0.9426	0.0809	0.7596	0.025*	
C21	0.90692 (19)	0.01333 (6)	0.81253 (18)	0.0235 (3)	
H21A	1.0037	0.0064	0.8656	0.028*	
C22	0.7969 (2)	−0.01989 (6)	0.7946 (2)	0.0268 (4)	
C23	0.6518 (2)	−0.01129 (7)	0.7168 (2)	0.0320 (4)	
H23A	0.5797	−0.0346	0.7062	0.038*	
C24	0.61669 (19)	0.03317 (7)	0.6547 (2)	0.0276 (4)	
H24A	0.5195	0.0398	0.6021	0.033*	
C25	0.65853 (18)	0.10922 (6)	0.34114 (17)	0.0192 (3)	
C26	0.5758 (2)	0.11696 (7)	0.19626 (19)	0.0267 (3)	
H26A	0.4805	0.1293	0.1751	0.032*	
C27	0.6368 (2)	0.10606 (7)	0.0851 (2)	0.0304 (4)	
H27A	0.5812	0.1110	−0.0107	0.037*	
C28	0.7796 (2)	0.08782 (6)	0.1134 (2)	0.0269 (4)	
H28A	0.8191	0.0805	0.0377	0.032*	
C29	0.86119 (19)	0.08080 (6)	0.25647 (18)	0.0216 (3)	
H29A	0.9571	0.0691	0.2768	0.026*	
C30	0.80235 (17)	0.09098 (5)	0.37050 (18)	0.0193 (3)	
H30A	0.8583	0.0857	0.4660	0.023*	
C31	0.5017 (2)	0.26415 (6)	0.4675 (2)	0.0254 (3)	
H31A	0.5007	0.2979	0.4539	0.038*	
H31B	0.4851	0.2486	0.3753	0.038*	
H31C	0.5961	0.2547	0.5307	0.038*	

### Atomic displacement parameters (Å^2^)

**Table:**

	*U*^11^	*U*^22^	*U*^33^	*U*^12^	*U*^13^	*U*^23^
Cl1	0.0240 (2)	0.0383 (3)	0.0390 (3)	0.00459 (17)	0.01745 (18)	−0.00127 (19)
F1	0.0510 (8)	0.0263 (6)	0.0492 (7)	0.0099 (5)	0.0267 (6)	0.0157 (5)
N1	0.0244 (6)	0.0182 (6)	0.0218 (6)	0.0019 (5)	0.0085 (5)	0.0027 (5)
N2	0.0189 (6)	0.0219 (6)	0.0206 (6)	0.0059 (5)	0.0070 (5)	0.0023 (5)
N3	0.0203 (6)	0.0259 (7)	0.0159 (6)	0.0081 (5)	0.0057 (5)	0.0012 (5)
C1	0.0216 (7)	0.0195 (7)	0.0187 (7)	0.0014 (5)	0.0072 (6)	0.0028 (5)
C2	0.0222 (7)	0.0161 (7)	0.0218 (7)	0.0020 (5)	0.0075 (6)	0.0007 (5)
C3	0.0319 (9)	0.0167 (7)	0.0335 (9)	0.0054 (6)	0.0145 (7)	0.0009 (6)
C4	0.0307 (9)	0.0219 (8)	0.0334 (9)	0.0078 (7)	0.0134 (7)	−0.0018 (7)
C5	0.0207 (7)	0.0267 (8)	0.0252 (8)	0.0046 (6)	0.0097 (6)	−0.0012 (6)
C6	0.0200 (7)	0.0202 (7)	0.0244 (7)	0.0023 (6)	0.0085 (6)	0.0005 (6)
C7	0.0175 (6)	0.0156 (6)	0.0196 (7)	0.0026 (5)	0.0061 (5)	0.0004 (5)
C8	0.0170 (6)	0.0159 (6)	0.0181 (6)	0.0012 (5)	0.0048 (5)	−0.0009 (5)
C9	0.0180 (6)	0.0176 (6)	0.0166 (6)	0.0037 (5)	0.0054 (5)	0.0014 (5)
C10	0.0160 (6)	0.0142 (6)	0.0231 (7)	−0.0003 (5)	0.0070 (5)	−0.0025 (5)
C11	0.0202 (7)	0.0220 (7)	0.0231 (7)	−0.0013 (6)	0.0058 (6)	−0.0048 (6)
C12	0.0242 (8)	0.0200 (7)	0.0365 (9)	−0.0059 (6)	0.0117 (7)	−0.0105 (7)
C13	0.0268 (8)	0.0172 (7)	0.0422 (10)	−0.0036 (6)	0.0162 (8)	−0.0012 (7)
C14	0.0259 (8)	0.0209 (7)	0.0288 (8)	−0.0012 (6)	0.0115 (7)	0.0039 (6)
C15	0.0211 (7)	0.0181 (7)	0.0229 (7)	−0.0018 (6)	0.0091 (6)	−0.0020 (6)
C16	0.0171 (6)	0.0179 (7)	0.0196 (7)	0.0021 (5)	0.0065 (5)	0.0009 (5)
C17	0.0227 (7)	0.0287 (8)	0.0171 (7)	0.0083 (6)	0.0051 (6)	−0.0009 (6)
C18	0.0174 (6)	0.0227 (7)	0.0175 (7)	0.0032 (5)	0.0043 (5)	−0.0001 (6)
C19	0.0188 (7)	0.0231 (7)	0.0176 (7)	0.0027 (6)	0.0072 (5)	0.0009 (5)
C20	0.0200 (7)	0.0234 (7)	0.0184 (7)	0.0021 (6)	0.0053 (6)	−0.0001 (6)
C21	0.0247 (8)	0.0261 (8)	0.0199 (7)	0.0054 (6)	0.0064 (6)	0.0030 (6)
C22	0.0352 (9)	0.0229 (8)	0.0267 (8)	0.0055 (7)	0.0157 (7)	0.0071 (6)
C23	0.0297 (9)	0.0296 (9)	0.0397 (10)	−0.0053 (7)	0.0146 (8)	0.0029 (8)
C24	0.0199 (7)	0.0304 (9)	0.0320 (9)	−0.0005 (7)	0.0066 (7)	0.0016 (7)
C25	0.0217 (7)	0.0191 (7)	0.0185 (7)	0.0020 (5)	0.0086 (6)	−0.0007 (5)
C26	0.0256 (8)	0.0335 (9)	0.0216 (8)	0.0065 (7)	0.0076 (6)	0.0012 (7)
C27	0.0329 (9)	0.0391 (10)	0.0201 (8)	0.0052 (8)	0.0088 (7)	0.0013 (7)
C28	0.0338 (9)	0.0268 (8)	0.0256 (8)	0.0018 (7)	0.0170 (7)	−0.0025 (7)
C29	0.0239 (7)	0.0179 (7)	0.0261 (8)	0.0006 (6)	0.0122 (6)	−0.0013 (6)
C30	0.0193 (7)	0.0179 (7)	0.0219 (7)	0.0010 (5)	0.0082 (6)	−0.0009 (5)
C31	0.0261 (8)	0.0247 (8)	0.0291 (8)	0.0012 (6)	0.0137 (7)	0.0054 (7)

### Geometric parameters (Å, °)

**Table:**

Cl1—C5	1.7429 (17)	C14—H14A	0.9300
F1—C22	1.363 (2)	C15—H15A	0.9300
N1—C1	1.319 (2)	C16—C17	1.509 (2)
N1—C2	1.372 (2)	C17—C18	1.536 (2)
N2—C16	1.281 (2)	C17—H17A	0.9700
N2—N3	1.4062 (18)	C17—H17B	0.9700
N3—C25	1.4009 (19)	C18—C19	1.511 (2)
N3—C18	1.480 (2)	C18—H18A	0.9800
C1—C9	1.432 (2)	C19—C20	1.389 (2)
C1—C31	1.503 (2)	C19—C24	1.399 (2)
C2—C3	1.417 (2)	C20—C21	1.398 (2)
C2—C7	1.421 (2)	C20—H20A	0.9300
C3—C4	1.366 (2)	C21—C22	1.372 (3)
C3—H3A	0.9300	C21—H21A	0.9300
C4—C5	1.411 (3)	C22—C23	1.380 (3)
C4—H4A	0.9300	C23—C24	1.387 (3)
C5—C6	1.369 (2)	C23—H23A	0.9300
C6—C7	1.418 (2)	C24—H24A	0.9300
C6—H6A	0.9300	C25—C30	1.402 (2)
C7—C8	1.427 (2)	C25—C26	1.404 (2)
C8—C9	1.384 (2)	C26—C27	1.385 (2)
C8—C10	1.493 (2)	C26—H26A	0.9300
C9—C16	1.486 (2)	C27—C28	1.393 (3)
C10—C15	1.397 (2)	C27—H27A	0.9300
C10—C11	1.402 (2)	C28—C29	1.384 (3)
C11—C12	1.393 (2)	C28—H28A	0.9300
C11—H11A	0.9300	C29—C30	1.392 (2)
C12—C13	1.386 (3)	C29—H29A	0.9300
C12—H12A	0.9300	C30—H30A	0.9300
C13—C14	1.387 (3)	C31—H31A	0.9600
C13—H13A	0.9300	C31—H31B	0.9600
C14—C15	1.393 (2)	C31—H31C	0.9600
			
C1—N1—C2	118.69 (14)	C16—C17—H17A	111.4
C16—N2—N3	108.31 (13)	C18—C17—H17A	111.4
C25—N3—N2	116.45 (12)	C16—C17—H17B	111.4
C25—N3—C18	121.92 (13)	C18—C17—H17B	111.4
N2—N3—C18	111.12 (12)	H17A—C17—H17B	109.3
N1—C1—C9	122.37 (14)	N3—C18—C19	113.35 (13)
N1—C1—C31	117.34 (14)	N3—C18—C17	101.44 (12)
C9—C1—C31	120.26 (14)	C19—C18—C17	112.60 (13)
N1—C2—C3	117.73 (14)	N3—C18—H18A	109.7
N1—C2—C7	122.82 (14)	C19—C18—H18A	109.7
C3—C2—C7	119.45 (15)	C17—C18—H18A	109.7
C4—C3—C2	120.78 (16)	C20—C19—C24	118.88 (16)
C4—C3—H3A	119.6	C20—C19—C18	120.24 (14)
C2—C3—H3A	119.6	C24—C19—C18	120.81 (14)
C3—C4—C5	119.22 (15)	C19—C20—C21	120.77 (16)
C3—C4—H4A	120.4	C19—C20—H20A	119.6
C5—C4—H4A	120.4	C21—C20—H20A	119.6
C6—C5—C4	122.13 (15)	C22—C21—C20	118.24 (16)
C6—C5—Cl1	120.01 (14)	C22—C21—H21A	120.9
C4—C5—Cl1	117.86 (12)	C20—C21—H21A	120.9
C5—C6—C7	119.37 (15)	F1—C22—C21	118.29 (17)
C5—C6—H6A	120.3	F1—C22—C23	118.68 (17)
C7—C6—H6A	120.3	C21—C22—C23	123.02 (17)
C6—C7—C2	119.04 (14)	C22—C23—C24	117.98 (17)
C6—C7—C8	123.17 (14)	C22—C23—H23A	121.0
C2—C7—C8	117.77 (13)	C24—C23—H23A	121.0
C9—C8—C7	118.24 (14)	C23—C24—C19	121.11 (17)
C9—C8—C10	119.10 (13)	C23—C24—H24A	119.4
C7—C8—C10	122.63 (13)	C19—C24—H24A	119.4
C8—C9—C1	120.07 (14)	N3—C25—C30	120.43 (14)
C8—C9—C16	120.27 (14)	N3—C25—C26	120.27 (14)
C1—C9—C16	119.23 (13)	C30—C25—C26	119.21 (14)
C15—C10—C11	119.28 (14)	C27—C26—C25	119.55 (16)
C15—C10—C8	120.74 (13)	C27—C26—H26A	120.2
C11—C10—C8	119.67 (14)	C25—C26—H26A	120.2
C12—C11—C10	120.08 (16)	C26—C27—C28	121.60 (17)
C12—C11—H11A	120.0	C26—C27—H27A	119.2
C10—C11—H11A	120.0	C28—C27—H27A	119.2
C13—C12—C11	120.18 (16)	C29—C28—C27	118.55 (15)
C13—C12—H12A	119.9	C29—C28—H28A	120.7
C11—C12—H12A	119.9	C27—C28—H28A	120.7
C12—C13—C14	120.02 (16)	C28—C29—C30	121.20 (16)
C12—C13—H13A	120.0	C28—C29—H29A	119.4
C14—C13—H13A	120.0	C30—C29—H29A	119.4
C13—C14—C15	120.30 (16)	C29—C30—C25	119.88 (15)
C13—C14—H14A	119.8	C29—C30—H30A	120.1
C15—C14—H14A	119.8	C25—C30—H30A	120.1
C14—C15—C10	120.02 (15)	C1—C31—H31A	109.5
C14—C15—H15A	120.0	C1—C31—H31B	109.5
C10—C15—H15A	120.0	H31A—C31—H31B	109.5
N2—C16—C9	123.64 (14)	C1—C31—H31C	109.5
N2—C16—C17	113.94 (13)	H31A—C31—H31C	109.5
C9—C16—C17	122.42 (13)	H31B—C31—H31C	109.5
C16—C17—C18	101.86 (12)		
			
C16—N2—N3—C25	−158.10 (14)	C8—C10—C15—C14	−170.26 (14)
C16—N2—N3—C18	−12.48 (18)	N3—N2—C16—C9	−178.89 (14)
C2—N1—C1—C9	−0.2 (2)	N3—N2—C16—C17	0.53 (19)
C2—N1—C1—C31	−178.48 (15)	C8—C9—C16—N2	114.72 (18)
C1—N1—C2—C3	178.56 (16)	C1—C9—C16—N2	−72.8 (2)
C1—N1—C2—C7	−1.5 (2)	C8—C9—C16—C17	−64.7 (2)
N1—C2—C3—C4	−179.70 (17)	C1—C9—C16—C17	107.78 (18)
C7—C2—C3—C4	0.4 (3)	N2—C16—C17—C18	10.63 (19)
C2—C3—C4—C5	−0.8 (3)	C9—C16—C17—C18	−169.94 (14)
C3—C4—C5—C6	0.3 (3)	C25—N3—C18—C19	−77.43 (18)
C3—C4—C5—Cl1	179.96 (15)	N2—N3—C18—C19	139.13 (13)
C4—C5—C6—C7	0.5 (3)	C25—N3—C18—C17	161.61 (15)
Cl1—C5—C6—C7	−179.14 (13)	N2—N3—C18—C17	18.17 (17)
C5—C6—C7—C2	−0.8 (2)	C16—C17—C18—N3	−16.11 (16)
C5—C6—C7—C8	177.67 (15)	C16—C17—C18—C19	−137.59 (14)
N1—C2—C7—C6	−179.49 (15)	N3—C18—C19—C20	132.50 (15)
C3—C2—C7—C6	0.4 (2)	C17—C18—C19—C20	−113.06 (16)
N1—C2—C7—C8	1.9 (2)	N3—C18—C19—C24	−50.6 (2)
C3—C2—C7—C8	−178.16 (15)	C17—C18—C19—C24	63.8 (2)
C6—C7—C8—C9	−179.16 (15)	C24—C19—C20—C21	−0.2 (2)
C2—C7—C8—C9	−0.6 (2)	C18—C19—C20—C21	176.76 (14)
C6—C7—C8—C10	−1.0 (2)	C19—C20—C21—C22	0.1 (2)
C2—C7—C8—C10	177.52 (14)	C20—C21—C22—F1	178.66 (14)
C7—C8—C9—C1	−0.9 (2)	C20—C21—C22—C23	0.0 (3)
C10—C8—C9—C1	−179.15 (14)	F1—C22—C23—C24	−178.82 (16)
C7—C8—C9—C16	171.44 (14)	C21—C22—C23—C24	−0.2 (3)
C10—C8—C9—C16	−6.8 (2)	C22—C23—C24—C19	0.2 (3)
N1—C1—C9—C8	1.4 (2)	C20—C19—C24—C23	0.0 (3)
C31—C1—C9—C8	179.67 (15)	C18—C19—C24—C23	−176.91 (16)
N1—C1—C9—C16	−171.04 (15)	N2—N3—C25—C30	155.63 (15)
C31—C1—C9—C16	7.2 (2)	C18—N3—C25—C30	14.0 (2)
C9—C8—C10—C15	114.82 (17)	N2—N3—C25—C26	−27.9 (2)
C7—C8—C10—C15	−63.3 (2)	C18—N3—C25—C26	−169.55 (16)
C9—C8—C10—C11	−58.7 (2)	N3—C25—C26—C27	−175.89 (17)
C7—C8—C10—C11	123.13 (16)	C30—C25—C26—C27	0.6 (3)
C15—C10—C11—C12	−1.4 (2)	C25—C26—C27—C28	−0.5 (3)
C8—C10—C11—C12	172.27 (14)	C26—C27—C28—C29	−0.3 (3)
C10—C11—C12—C13	−1.8 (2)	C27—C28—C29—C30	0.9 (3)
C11—C12—C13—C14	3.1 (3)	C28—C29—C30—C25	−0.8 (2)
C12—C13—C14—C15	−1.2 (3)	N3—C25—C30—C29	176.51 (15)
C13—C14—C15—C10	−2.1 (2)	C26—C25—C30—C29	0.0 (2)
C11—C10—C15—C14	3.3 (2)		

### Hydrogen-bond geometry (Å, °)

**Table:**

Cg1 and Cg2 are the centroids of the N1/C1/C2/C7–C9 and C10–C15 rings, respectively.

**Table:**

*D*—H···*A*	*D*—H	H···*A*	*D*···*A*	*D*—H···*A*
C15—H15A···N1^i^	0.93	2.57	3.493 (2)	173
C17—H17A···Cg1	0.97	2.86	3.6307 (19)	137
C31—H31B···Cg2^ii^	0.96	2.86	3.584 (2)	133

Symmetry codes: (i) *x*, −*y*+1/2, *z*+1/2;  (ii) *x*, −*y*−1/2, *z*−3/2.
